# Porous polymer coatings as substrates for the formation of high-fidelity micropatterns by quill-like pens

**DOI:** 10.3762/bjnano.4.44

**Published:** 2013-06-19

**Authors:** Michael Hirtz, Marcus Lyon, Wenqian Feng, Andrea E Holmes, Harald Fuchs, Pavel A Levkin

**Affiliations:** 1 Institute of Nanotechnology (INT) and Karlsruhe Nano Micro Facility (KNMF), Karlsruhe Institute of Technology (KIT), Germany; 2 Department of Chemistry, Doane College, Crete, Nebraska, and the Center for Nanohybrid Functional Materials (CNFM), University of Nebraska-Lincoln, USA; 3 Institute of Toxicology and Genetics (ITG), Karlsruhe Institute of Technology (KIT), Germany; 4 Physical Institute and Center for Nanotechnology (CeNTech), University of Münster, Germany; 5 Applied Physical Chemistry, Heidelberg University, Germany

**Keywords:** microarrays, microscale printing, microspotting, polymeric porous support, polymethacrylate, quill-like pens

## Abstract

We explored the potentials of microarray printing using quill-like microcantilevers onto solid supports that are typically used in microspot printing, including paper, polymeric nitrocellulose and nylon membranes. We compared these membranes with a novel porous poly(2-hydroxyethyl methacrylate-*co*-ethylene dimethacrylate) support (HEMA) with narrow pore size distribution in the 150 nm range, which demonstrated advantages in pattern definition, spot homogeneity, and consistent spot delivery of different dyes (phloxine B and bromophenol blue) with diameters of several micrometres. The bromophenol blue arrays on HEMA support were used to detect the presence of bovine serum albumin (BSA). In the presence of BSA, the fluorescence spectrum observed from the bromophenol blue microarray exhibited a significant red shift of the maximum emission wavelength. Our results show that the porous HEMA substrates can improve the fidelity and quality of microarrays prepared by using the quill-like microcantilevers. The presented method sets the stage for further studies using chemical and biochemical recognition elements, along with colorimetric and fluorometric sensors that can be spotted by this method onto flat porous polymer substrates.

## Introduction

Microarrays are of immense importance in many fields of biological research (e.g., genomics, proteomics, and cell analysis) and medical applications in diagnostics such as the detection of pathogens or antibodies. Nitrocellulose films and nylon membranes are widely used as carriers for microarrays, usually with spot sizes in the range of 100 to 500 µm, easily accessible for current inkjet and spotting techniques [1]. For scales of 100 µm and above, even plain paper was proposed as an inexpensive substrate for microfluidic devices [2–3]. However, when downsizing microarrays to the lower micrometer range with spot features in the range of a few tens of micrometres, the intrinsic granularity and broad pore size distribution of these substrates impairs pattern fidelity, quality and reproducibility. In addition, porous substrates that feature a large porous morphology are usually nontransparent due to the extensive light scattering, which reduces the sensitivity of readout systems utilizing such substrates.

Recently we introduced a method for the preparation of a porous biocompatible polymer coating on a solid substrate, using in situ free radical polymerization of methacrylate monomers in the presence of porogens [4]. Porous poly(2-hydroxyethyl methacrylate-*co*-ethylene dimethacrylate, HEMA) was shown to possess very high hydrophilicity due to the combination of the porous structure with the hydrophilic nature of the 2-hydroxyethyl methacrylate used as a monomer [5]. The small size and narrow size distribution of both pores and polymer globules (about 20–200 nm) resulted in high transparency of the polymer in the wetted state [4]. Such porous HEMA substrates were used for creating superhydrophilic–superhydrophobic micropatterned surfaces for cell-patterning [6] and cell-screening applications [7–8].

Here, we present an approach for the formation of high-fidelity microarrays of three-dimensional 20–50 micrometer sized spots inside a HEMA film, using quill-like microcantilevers. In contrast to nonporous substrates, porous films allow for the noncovalent infiltration of fluorescent sensors or dyes that can accommodate a greater volume and increased surface area for analyte binding. This should enhance sensitivity and yield a more reliable read out due to higher signal strength and less potential for cross contamination of sensors due to bleeding or trailing. The increased transparency of the porous HEMA substrates also allows for detection in transmission mode, which increases the versatility of this technique. Combined, these advantages of the porous HEMA substrates over plain surfaces and other porous substrates with larger pores or broader pore size distribution make them ideal candidates for creating high-fidelity micropatterns and microarrays by using the quill-like pens.

## Results and Discussion

### Pattern generation

**Figure 1:**
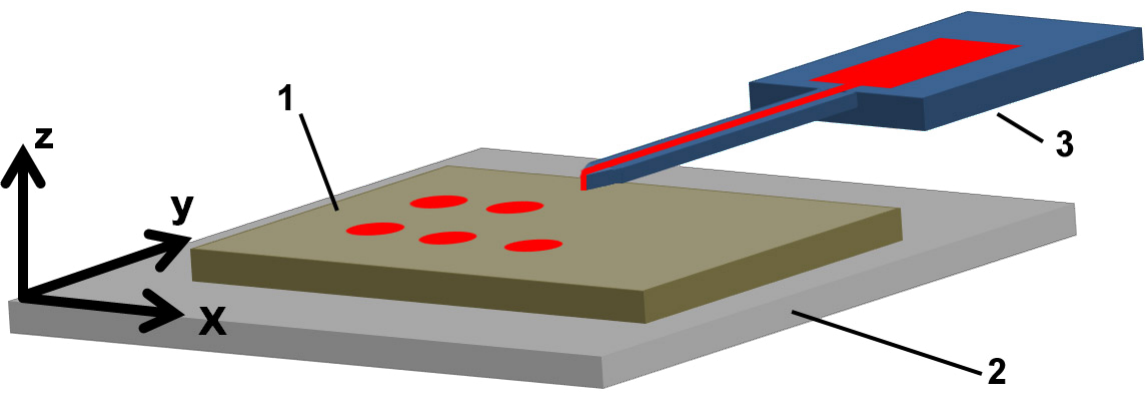
Dye delivery by microchannel cantilever. The substrate (1) is placed on the stage (2), which can be actuated with a precision of less than 100 nm in the *x*-, *y*-, and *z*-direction by piezoelectric actuators. By raising the stage in the *z*-direction the substrate can be brought into contact with the apex of the microchannel cantilever of the SPT (3) on which the dye solution reservoir is located.

The microarrays were fabricated by spotting the dye solution with quill-like microchannel cantilevers, called surface patterning tools (SPTs) [9], attached to a dip-pen nanolithography (DPN) platform (NLP 2000, NanoInk, USA) for precise control in *x*- *y*- and *z*-direction (Figure 1). After filling of the reservoir on the SPT with the dye solution, it is brought into contact with the substrate surface for a defined dwell time to allow a flow to the substrate by capillary forces. The SPT is retracted and moved to the next spotting position. The process of relocation, contacting and retracting is repeated until the desired spot features are created. The writing procedure can be relatively fast: our standard pattern of 100 spots arranged in a square with pitch of 50 µm (yielding a patterned area of 500 × 500 µm^2^) with a dwell time of 0.5 s was written with a single cantilever in about a minute. However, technically the writing speed can be increased by the use of cantilever arrays, with the added option of intrinsic multiplexing [10].

### Comparison of substrates

**Figure 2:**
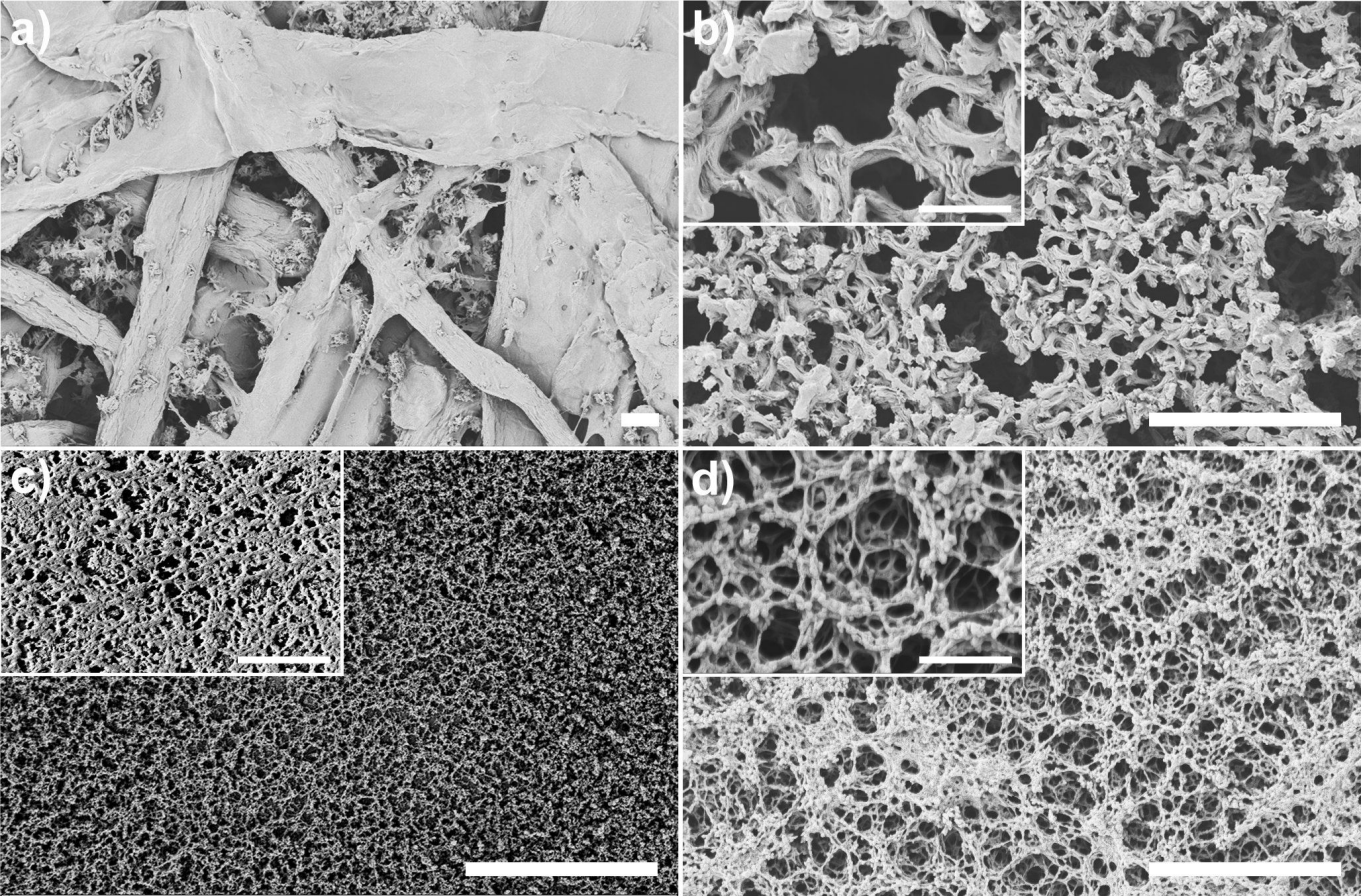
SEM images of the (a) plain paper, (b) nylon membrane, (c) HEMA polymer and (d) nitrocellulose substrates used for patterning. The scale bars respresent 10 µm in the main images and 2 µm in the insets.

First, we compared four different substrate systems (i.e., plain paper, nylon membrane, nitrocellulose, and a HEMA porous polymer film, see Experimental section for exact type and suppliers) for their capability to serve as platforms for the microarrays generated by spotting with SPTs. Since porosity was considered as a key aspect for pattern fidelity, scanning electron microscopy (SEM) images of the different substrates were recorded to estimate pore size distribution and morphology (Figure 2).

**Figure 3:**
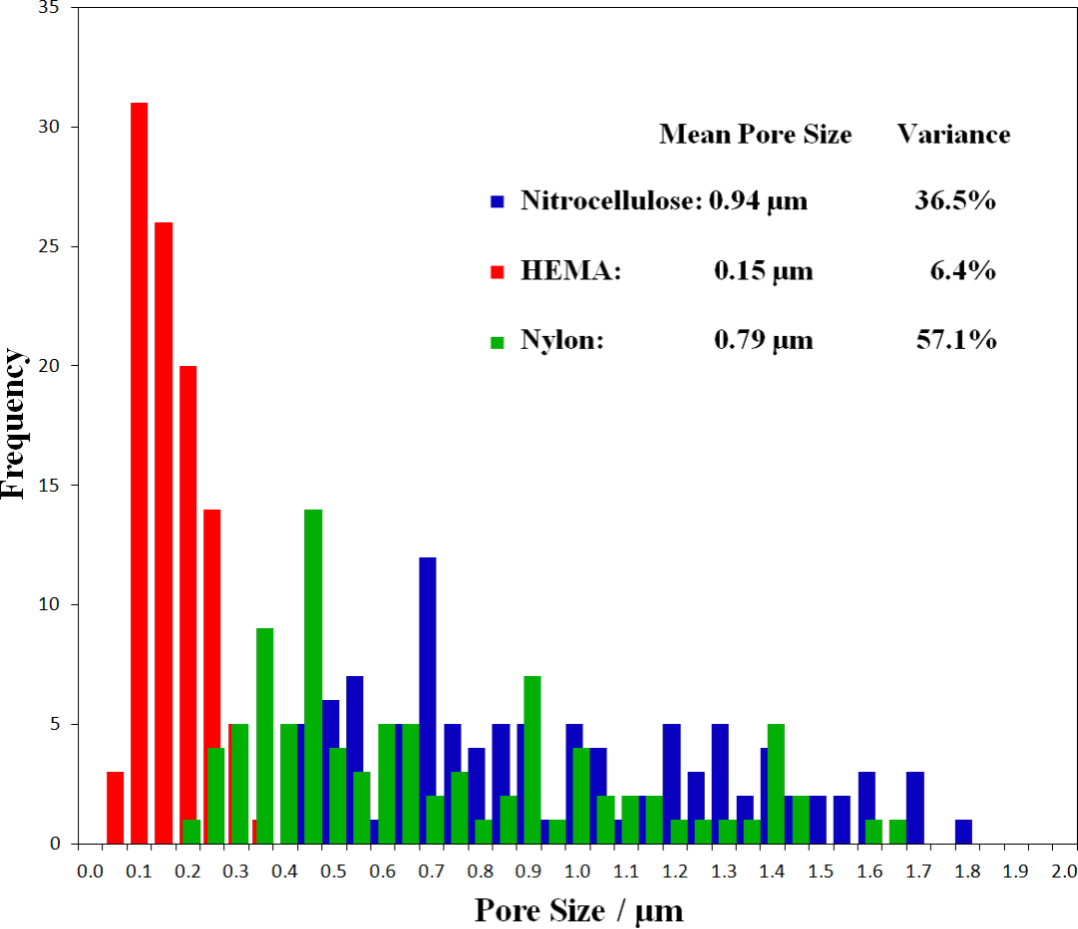
Pore size distribution based on SEM micrographs for the three different porous substrates, measured over 100 pores for each substrate. The mean pore size and variance for each porous substrate is given in the inset.

The plain paper is of fibrous morphology, with dense fibres in the width range of 10 to 50 µm (Figure 2a) and gaps of about 50 µm. The microporous nylon membrane has a nominal pore size of 0.45 µm and is positively charged by quaternary ammonium groups (supplier specification). The average pore size obtained from SEM amounts to (0.79 ± 0.57) µm and is compatible with the specification within one standard deviation, but the overall pore size distribution seems broad with many pores sizing up to almost 2 µm (Figure 2b). The porous HEMA substrate shows a highly porous (60% porosity based on the prepolymer mixture) structure of interconnected polymer globules, with the size of pores and globules being in the range of (0.15 ± 0.06) µm (Figure 2c). The thickness of the film is about 12.5 µm as adjusted by the spacers used in the preparation process. The nitrocellulose membranes with a thickness of 10 to 15 µm (supplier specification) is also highly porous with the average pore size estimated from SEM being (0.94 ± 0.37) µm. Figure 3 presents a histogram of the pore size distribution in the three different porous substrates. The HEMA substrate shows a much narrower distribution and smaller average pore size compared to the nylon membrane and nitrocellulose film. The paper substrate was not included in the plot due to its significantly larger pores and pore size distribution.

**Figure 4:**
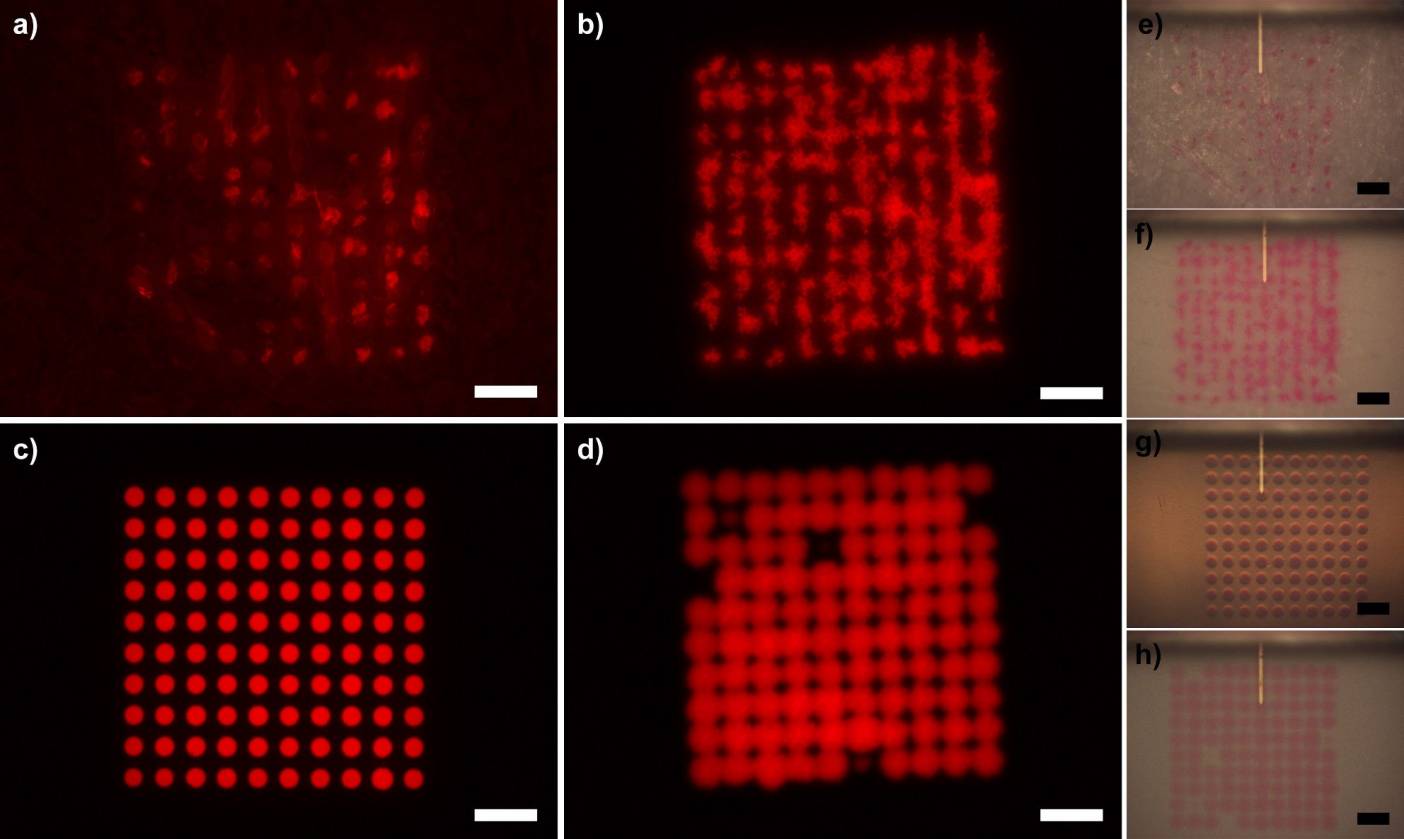
Comparison of printed phloxine B solution on different substrates. Fluorescence microscopy images of the printed solution on (a) paper, (b) nylon, (c) porous HEMA polymer film, and (d) nitrocellulose. Corresponding in situ bright-field images with delivery microchannel cantilever still in place for (e) paper, (f) nylon, (g) porous HEMA polymer film, and (h) nitrocellulose, respectively. Scale bars equal 100 µm.

As shown in Figure 4, four different substrates were tested as platforms for the microarray spotting using SPTs. A pattern of 10 × 10 spots with a 50 µm pitch and dwell time of 0.5 s was written on each of the substrates by using a 10 mM solution of phloxine B in isopropanol mixed with 30 vol % glycerol (87% in water) to prevent drying of the dye solution in the SPT reservoir.

Bright field and fluorescent microscopy images demonstrate huge differences in the patterning outcome for the different substrates. Plain paper (Figure 4a and Figure 4e) does not consistently take up phloxine B from the SPT, presumably in part because of a large surface roughness (that might have prevented the SPT from touching the surface in some places) and because of inhomogeneous wetting behaviour due to the fibrous structure of the paper, as seen by sometimes brighter and sometimes fainter features. Overall the rough surface structure prevents clear homogenous patterning of phloxine B, and spreading is observed along the fibres that are tens to hundreds of micrometers long (see Supporting Information File 1, Figure S1 for combined bright field and fluorescence images). This behaviour is consistent with the observation that hydrophobic barriers have to be on the order of at least 200 µm to be effective in paper-based microfluidics [3]. Patterning on nylon membranes (Figure 4b and Figure 4f) shows a uniform wetting behaviour over the whole substrate area (visible by equal fluorescence intensity in the different features). However, similar distortions, as seen on the paper substrate, caused by the solution trailing along the nylon fibres of the membrane were observed. In both cases, this trailing is on the order of few tens of micrometers, which may be acceptable for spot sizes in the range of hundreds of micrometers as in conventional microarrays. However, patterning of microarrays with spot sizes in the lower micron range on such substrates becomes impractical. The porous nitrocellulose performs much better with regard to pattern fidelity (Figure 4d and Figure 4h), reflecting the fact that it is more homogeneous with smaller fibres and pore organisation as compared to paper and nylon membrane. However, compared with the pattern on porous HEMA (Figure 4c and Figure 4g) the pattern on nitrocellulose still has a much lower definition, with more diffuse spot edges and some missing features. The pattern on porous HEMA has by far the sharpest spot edges and most homogeneous spot distribution within the features.

### Characterization of printing on porous HEMA substrate

**Figure 5:**
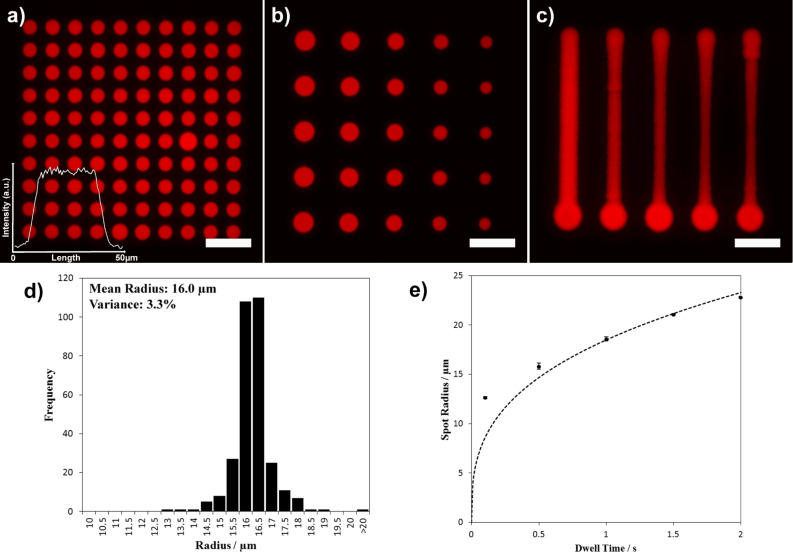
Time dependence and homogeneity of microprinting on porous polymer HEMA films. Fluorescent micrographs of (a) a spot pattern with 0.5 s dwell time, the inset shows the intensity profile of a typical spot, (b) spot pattern with varying dwell time (from left column to right 2.0 s, 1.5 s, 1.0 s, 0.5 s, and 0.1 s, respectively), and (c) lines written with different speeds (from left to right 50 µm/s, 100 µm/s, 150 µm/s, 200 µm/s, and 250 µm/s, respectively). Lines were written from bottom to top. All scale bars equal 100 µm. A histogram of the spot radius of the image in (a) combined with two more patterns generated under the same conditions for more statistics is given in (d). The dependence of spot radius on the dwell time as derived from the image in (c) is given in (e), dashed line denotes a plot of *y* = *A*·*x*^1/3^ with *A* = 18.5.

Several experiments on the porous HEMA were performed to evaluate reproducibility and tunability. Three spot patterns of 100 spots (0.5 s dwell time) are shown in Figure 5a with an average spot radius of (16.0 ± 0.7) µm. The size distribution is narrow (Figure 5d) and symmetrical, showing very good pattern reproducibility with variance in radius of only 3.3%. The intensity within a spot is very uniform, with fluctuations of around 3% (profile inset in Figure 5a).

The size of the spots can be tuned by varying the dwell time (Figure 5b). By increasing the dwell time from 0.1 s to 2.0 s the radius of the spot features grows from (12.6 ± 0.1) µm to (22.8 ± 0.1) µm as seen in Figure 5e. Theoretically, for a liquid being absorbed by a porous media from a point source one should expect a growth in radius with a dependence *r* ~ *t*^1/3^, denoted by the dashed line [11]. Our results deviate from this trend for the low dwell time of 0.1 s, which can be understood when taking into account that the theory postulates a point source for the liquid flow. In contrast, the SPT used in our experiments has a finite apex opening in the low micrometre range, therefore generating bigger spots than may be theoretically expected for short dwell times.

By moving the SPT over the substrate while keeping contact, line patterns can be generated with the line width depending on the speed of movement (Figure 5c). The lines were written from bottom to top with speeds of 50 µm/s (left line) up to 250 µm/s (right line) yielding line widths from about 22 µm to 12 µm width. The start point of the line is demarked by a bigger spot, since the DPN instrument pauses shortly after contacting the SPT with the surface before drawing the line. Especially for the higher velocity lines, there is a widening visible at the start and end of the lines. This is caused by the acceleration and deceleration, respectively, of the piezo positioning table of the instrument before reaching the target speed or when slowing down before the end of a line.

For microarray printing, the inherent three-dimensional porosity is advantageous over flat substrates, such as glass, because subsurface regions under a spot can act as a binding area for the spotted sensor solution and later on for the target analyte.

In our experiments, five times longer exposure times were needed for the plain paper and nylon substrates in comparison to the HEMA substrate to reach equal fluorescence intensity in the images (50 ms for plain paper and nylon membrane compared to 10 ms to HEMA, the nitrocellulose falls in between with 40 ms). This could be explained by the larger surface area and better transparency of the porous HEMA in comparison to the other substrates. The three-dimensional porous structure, improved transparency, and the large specific surface area can lead to considerably higher sensitivity when compared to substrates with the same surface area (see Supporting Information File 1 for comparison to a flat glass substrate, Figure S2).

### Detection of BSA with bromophenol blue pattern

**Figure 6:**
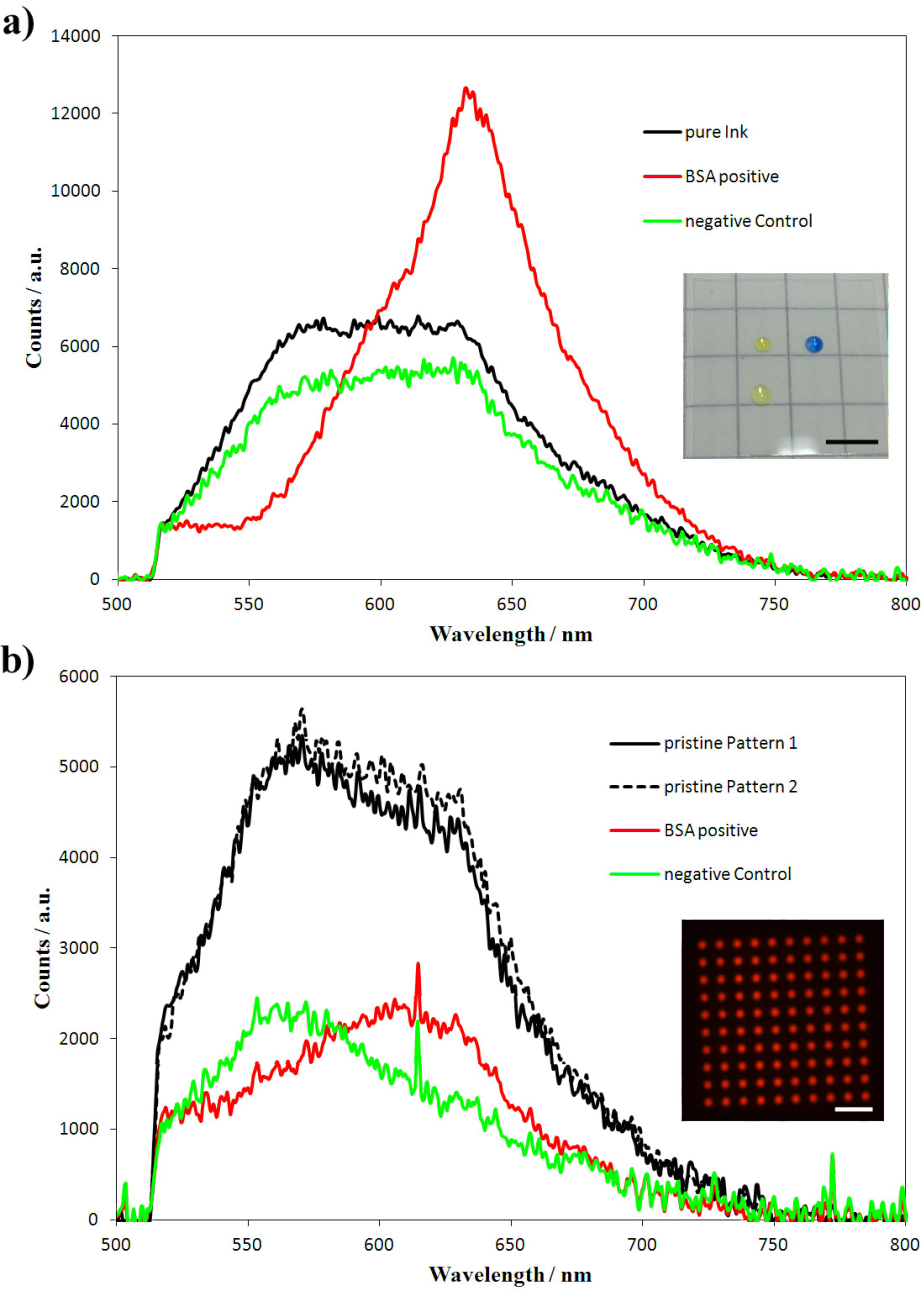
Fluorescence spectra of (a) macroscopic droplets of pure bromophenol blue dye solution (black), dye solution admixed with BSA (red), and dye solution admixed with a BSA free solution (green) as negative control (inset shows a photograph of the macroscopic droplets on a cover glass, pure dye solution at the top left, BSA positive at the top right and negative control at the bottom left position; scale bar equals 5 mm) and (b) of the pristine bromophenol blue pattern after lithography (black) and after incubation with BSA solution (red) or a solution not containing BSA (green), respectively (inset shows a fluorescence microscopy image of one of the pristine bromophenol blue patterns, scale bar equals 100 µm).

We used the HEMA substrate as a platform for demonstrating a microarray sensor application using bromophenol blue as a sensor, which is a dye commonly used in lateral flow devices to detect proteins in biological samples [12–13]. After preparing 10 × 10 spot microarrays using bromophenol blue solution as shown in the inset of Figure 6b, the patterns were either incubated with a 0.5 µL droplet of an analyte solution containing bovine serum albumin (BSA) or a clear solution to give a negative control. Fluorescence spectra obtained on the samples prior to and after incubation are shown in Figure 6b. Additionally, Figure 6a gives the corresponding fluorescence spectra of macroscopic droplets of the dye solutions with and without the addition of BSA for comparison.

All spectra were normalized to have equal intensity at the filter cut-off wavelength of 510 nm. The spectra of the patterns in Figure 6b were multiplied by a factor of 2 to demonstrate the spectroscopic changes more clearly. Figure 6a shows the fluorescence spectra of macroscopic droplets as shown in the inset. The fluorescence spectrum of the bromophenol in a macroscopic droplet (black curve) shows a very pronounced shift to the red after addition of BSA containing analyte solution (red curve), while the spectrum shape remains unaltered on addition of a control solution containing no BSA (green curve). For the bromophenol blue microarrays we obtain similar results (Figure 6b). The spectra of two patterns are given in black; the green curve shows the spectrum after incubation with a test solution containing no BSA and the red curve after incubation with a test solution containing 10% BSA. Again a substantial bathochromic shift in fluorescence distinguishes the control sample from a sample incubated with BSA.

## Conclusion

Four different substrates were tested for low-micrometre microarray printing with quill-like microcantilevers. We demonstrated that the porous HEMA polymer films exhibited advantageous properties for creating micropatterns with feature sizes below 50 μm. We showed that the narrow pore size distribution as well as the small average pore size is crucial for achieving high pattern fidelity and reproducibility. Additionally, the three-dimensional morphology in nanoporous substrates like the HEMA polymer films presents a higher surface area for binding of analytes, potentially giving rise to increased sensitivity in sensor applications. The pattern formation by SPTs can be tuned by varying the dwell time, or in the case of line patterns, by using different writing speeds. The basic feasibility of the HEMA as substrate for microarray-based sensor applications was demonstrated by the detection of BSA with a bromophenol blue pattern. This avenue should be followed further by the generation of sensor arrays aiming at more than one analyte and preferably incorporation into microfluidic systems for the delivery of analyte solutions under controlled conditions. This would open the door for the miniaturization of array sensor platforms like DETECHIP [14–15] and make them less expensive and more compatible with in-field applications.

## Experimental

**Spotting Setup:** All patterns were written on a NLP 2000 system (NanoInk, USA) equipped with SPT pens (SPT-S-C10S, Bioforce Nanosciences). The SPTs were freshly plasma cleaned by oxygen plasma (10 sccm O_2_, 100 mTorr, 30 W for 2 min) prior to use. The SPT was mounted onto the tip holder by double-sided sticky tape, and the pen reservoir was filled with 1 µL of dye solution. The spotting took place at a relative humidity of 60% and with the sample stage tilted by 8° with respect to the SPT tip to minimize the chance of contact between the dye solution in the reservoir and sample. For all patterns, except for the pattern used for spot size versus dwell time analysis, a dwell time of 0.5 s was used.

**Substrates:** Plain paper (Black Label Zero 80 g/m^2^, Canon) and nylon membrane (Nytran SuPerCharge (SPC), Whatman) were cut down into pieces of about 1 × 1 cm^2^ before spotting. The nitrocellulose slides (FAST Slides, Whatman) were used as received. HEMA polymer films were prepared as follows by using the previously described procedure [7]:

Schott (Germany) Nexterion Glass B glass plates were activated in NaOH (1 M) for 1 h followed by 30 min in 1 M HCl. A set of glass slides was modified with 3-(trimethoxysilyl)propyl methacrylate (20% v/v in ethanol) for 1 h. Another set of activated glass slides was fluorinated with tridecafluoro-(1,1,2,2)-tetrahydrooctyltrichlorosilane in a vacuumed desiccator. In the next step, the polymerization mixture consisted of 2-hydroxyethyl methacrylate (24 wt %), ethylene dimethacrylate (16 wt %), 1-decanol (12 wt %), cyclohexanol (48 wt %), and 2,2-dimethoxy-2-phenylacetophenone (1 wt % with respect to monomers) was injected between fluorinated and modified glass slides separated by 12.5 μm thick strips of Teflon film (American Durafilm Co.), and irradiated for 15 min at 12 mW·cm^−2^ with a 260 nm UV light to form a hydrophilic porous polymer layer. Polymerization was carried out on an OAI Model 30 deep-UV collimated light source (San Jose, CA) fitted with an USHIO 500 W Hg-xenon lamp (Japan). Irradiation intensity was calibrated by using an OAI 360 UV power meter with a 260 nm probe head. Monomers were purchased from Sigma-Aldrich (Germany). Further details can be found in our previous papers [6–7].

**Dye and analyte solutions:** All chemicals were obtained from Sigma-Aldrich and used as received if not otherwise noted. Ultrapure water (18.2 MΩ·cm) for the solutions was obtained from an Arium water supply (Sartorius, Germany). Phloxine B solution: 10 mM phloxine B in isopropanol was mixed 7:3 with a glycerol stock solution of 87% glycerol in water. Bromophenol blue solution: 1.4 mg/mL bromophenol blue in water was mixed 7:3 with a glycerol stock solution of 87% glycerol in water. Analyte solution: A mix of 10% BSA in water and glycerol stock solution (87% in water) 7:3 was used as the analyte solution in the bromophenol blue experiments. As the negative control, a mix of pure water and glycerol solution (87% in water) 7:3 was used.

**Scanning electron microscopy (SEM) analysis:** The surface morphologies of the substrates were analysed by using a ZEISS Leo 1530 scanning electron microscope (Carl Zeiss NTS GmbH, Germany) after gold sputtering (20 nm) using the Balzers Union MED 10.

**Microscope setup:** All fluorescent microscopic images were obtained on an Eclipse 80i upright fluorescence microscope (Nikon) equipped with an Intensilight (Nikon) for illumination and a CoolSNAP HQ^2^ camera (Photometrics). Phloxine B patterns were observed in Texas Red filter (Nikon Y-2E/C) with exposure times ranging from 10 ms (HEMA substrates) over 40 ms (nitro cellulose substrate) to 50 ms (plain paper and nylon). The fluorescent spectra were recorded on the same microscope through an AHF F36-QLP filter (excitation: 415–455 nm, dichroic mirror: 510 nm, emission: long-pass 500 nm) with an Avaspec-2048 Spectrometer (Avantes). Recording time for the spectra of the macroscopic droplets and the pristine microarray patterns was 1 s; the spectra of the incubated patterns were recorded with 3 s exposure.

**Image analysis:** To obtain the size distribution of the spotted features, fluorescent microscopy images were analysed with ImageJ [16–17]. The images were first converted into black and white by a threshold filter, and then spot sizes were obtained by particle analyses. Particles smaller than 20 pixels were excluded from analysis to exclude noise-induced artefacts.

## Supporting Information

File 1 Figures S1 and S2.
