# Supplementary material for: Porous polymer coatings as substrates for the formation of high-fidelity micropatterns by quill-like pens
Source: Beilstein J Nanotechnol. 2013 Jun 19;4:377–84. doi: 10.3762/bjnano.4.44 (PMC3701425; doi:10.3762/bjnano.4.44)
Supplement: File 1 — Figures S1 and S2. [file Beilstein_J_Nanotechnol-04-377-s001.pdf]

Supporting Information

for

Porous polymer coatings as substrates for the formation of high-fidelity micropatterns by quill-like pens

Michael Hirtz*[1], Marcus Lyon[2], Wenqian Feng[3], Andrea E. Holmes[2], Harald Fuchs[1,4], and Pavel A. Levkin*[3,5]

Address: [1]Institute of Nanotechnology (INT) and Karlsruhe Nano Micro Facility (KNMF), Karlsruhe Institute of Technology (KIT), Germany, [2]Department of Chemistry, Doane College, Crete, Nebraska, and the Center for Nanohybrid Functional Materials (CNFM), University of Nebraska-Lincoln, USA, [3]Institute of Toxicology and Genetics (ITG), Karlsruhe Institute of Technology (KIT), Germany, [4]Physical Institute and Center for Nanotechnology (CeNTech), University of Münster, Germany and [5]Applied Physical Chemistry, Heidelberg University, Germany

Email: Michael Hirtz - michael.hirtz@kit.edu; Pavel Levkin - levkin@kit.edu

* Corresponding author

Figures S1 and S2

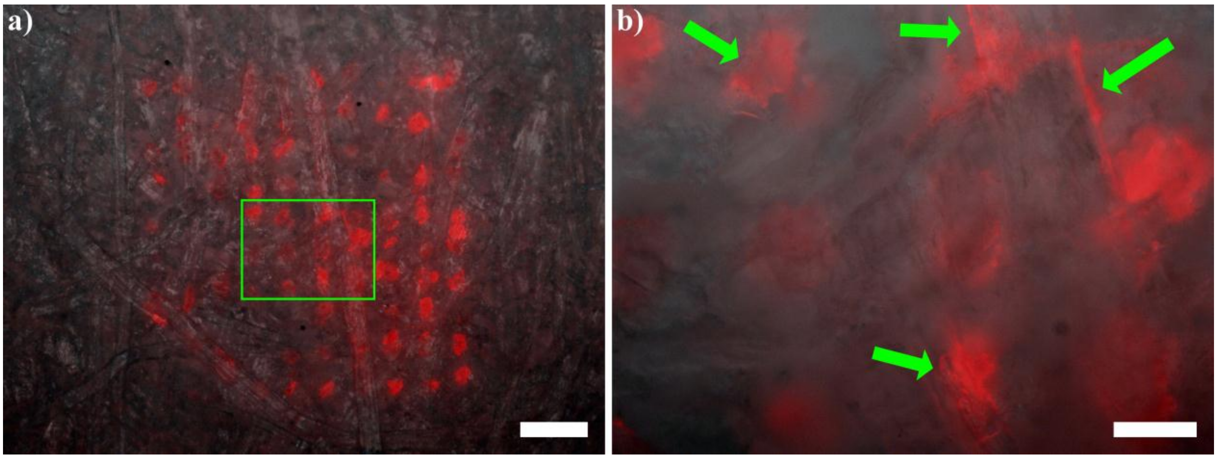

Figure S1: Combined bright-field and fluorescence microscopy images of the plain paper substrate after lithography. (a) shows an overview of the whole pattern, it can be observed that the distortion of the spots aligns along the direction of the paper fibres. Also it should be noted that the diameter of the paper fibres are itself in the same size range as the spotted dots. The scale bar equals 100 µm. (b) shows a close up of the green box marked in (a). Some instances of pronounced spreading along the paper fibres are marked by green arrows. The scale bar equals 25 µm.

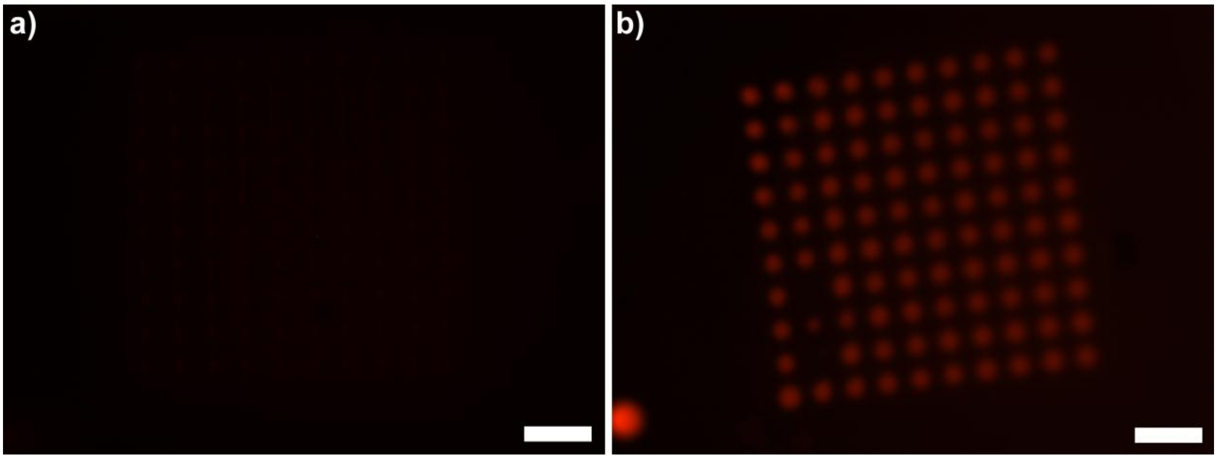

Figure S2: Comparative lithography on a flat glass substrate to obtain a monomolecular film pattern for comparison to surface-area enhancement in porous substrates; binding strategy as described elsewhere [1]. (a) molecular monolayer TAMRA-Azide pattern on flat glass and (b) bromophenol blue pattern on HEMA, both after incubation with water and under similar exposure conditions. All scale bars equal 100 µm.

References

1. Oberhansl, S.; Hirtz, M.; Lagunas, A.; Eritja, R.; Martinez, E.; Fuchs, H.; Samitier, J. *Small* **2012,** *8,* 541–545. doi:10.1002/smll.201101875
